# Minimally invasive determination of pancreatic ductal adenocarcinoma (PDAC) subtype by means of circulating cell‐free RNA

**DOI:** 10.1002/1878-0261.13747

**Published:** 2024-10-31

**Authors:** Martin Metzenmacher, Gregor Zaun, Marija Trajkovic‐Arsic, Phyllis Cheung, Timm M. Reissig, Hendrik Schürmann, Nils von Neuhoff, Grainne O'Kane, Stephanie Ramotar, Anna Dodd, Steven Gallinger, Alexander Muckenhuber, Jennifer J. Knox, Volker Kunzmann, Peter A. Horn, Jörg D. Hoheisel, Jens T. Siveke, Smiths S. Lueong

**Affiliations:** ^1^ Department of Medical Oncology, West German Cancer Center University Hospital Essen Germany; ^2^ German Cancer Consortium (DKTK), partner site Essen/Düsseldorf, a partnership between German Cabcer Research Center (DKFZ) and University Hospital Essen Germany; ^3^ Bridge Institute of Experimental Tumor Therapy (BIT) and Division of Solid Tumor Translational Oncology (DKTK), West German Cancer Center, University Hospital Essen University of Duisburg‐Essen Essen Germany; ^4^ Department of Pediatric Hematology and Oncology, Department for Pediatrics III University Hospital of Essen Germany; ^5^ PanCuRx Translational Research Initiative Ontario Institute for Cancer Research Toronto Canada; ^6^ Wallace McCain Centre for Pancreatic Cancer, Princess Margaret Cancer Centre University Health Network Toronto Canada; ^7^ Institute of Pathology Technical University of Munich Germany; ^8^ German Cancer Consortium (DKTK), Partner Site Munich Germany; ^9^ Department of Internal Medicine II, Medical Oncology, Comprehensive Cancer Center Mainfranken Würzburg University Hospital Würzburg Germany; ^10^ Institute for Transfusion Medicine University Hospital of Essen Germany; ^11^ Division of Functional Genome Analysis German Cancer Research Center (DKFZ) Heidelberg Germany

**Keywords:** cfRNA, liquid biopsy, PDAC, subtype, therapy

## Abstract

Pancreatic ductal adenocarcinoma (PDAC) comprises two clinically relevant molecular subtypes that are currently determined using tissue biopsies, which are spatially biased and highly invasive. We used whole transcriptome sequencing of 10 plasma samples with tumor‐informed subtypes, complemented by proteomic analysis for minimally invasive identification of PDAC subtype markers. Data were validated in independent large cohorts and correlated with treatment response and patient outcome. Differential transcript abundance analyses revealed 32 subtype‐specific, protein‐coding cell‐free RNA (cfRNA) transcripts. The subtype specificity of these transcripts was validated in two independent tissue cohorts comprising 195 and 250 cases, respectively. Three disease‐relevant cfRNA‐defined subtype markers (*DEGS1*, *KDELC1*, and *RPL23AP7*) that consistently associated with basal‐like tumors across all cohorts were identified. In both tumor and liquid biopsies, the overexpression of these markers correlated with poor survival. Moreover, elevated levels of the identified markers were linked to a poor response to systemic therapy and early relapse in resected patients. Our data indicate clinical applicability of cfRNA markers in determining tumor subtypes and monitoring disease recurrence.

## Graphical abstract

Subtype determination of the two clinically relevant molecular subtypes of pancreatic cancer has exclusively relied on tumor tissue samples, which are unfortunately not always available, require highly invasive procedures to obtain and, owing to tumor heterogeneity, provide limited results. In this proof‐of‐concept study, we deploy next‐generation sequencing and digital PCR to provide evidence for the clinical utility of cell‐free RNA for determination of PDAC subtypes.

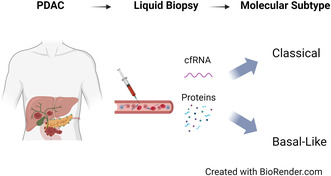

**Abbreviations**
C: copies per 20 μL reaction
cfRNA: cell‐free RNA
CI: confidence interval
ctDNA: circulating cell‐free tumor DNA
DDA: data‐dependent acquisition
ECM: extracellular matrix
EDTA: ethylenediaminetetraacetic acid
ELISA: Enzyme‐linked Immunosorbent Assay
EMT: epithelial‐to‐mesenchymal transition
EV: elution volume
FDR: false discovery rate
FFPE: formalin‐fixed paraffin‐embedded
FFX: FOLFIRINOX
g: acceleration size of the centrifuge
GnP: gemcitabine plus nab‐paclitaxel
HD: University of Heidelberg
HR: hazard ratio
IPMN: intraductal papillary mucinous neoplasms
ISV: cfRNA input volume
LC–MS/MS: Liquid Chromatography with tandem mass spectrometry
LFQ: label‐free quantification
m/z: mass‐to‐charge
maxIT: maximum injection time
MBR: match between runs
NCE: Normalized Collision EnergyT
PDAC: Pancreatic ductal adenocarcinoma
pH: potential of hydrogen
*Qm*
: *quasi‐mesenchymal*
RNA: Ribonucleic acid
rpm: runs per min
RT‐ddPCR: real‐time droplet digital PCR
TPV: total plasma/serum volume used of cfRNA isolation

## 1 Introduction

Pancreatic ductal adenocarcinoma (PDAC) stands as the most prevalent malignancy of the exocrine pancreas. With a disheartening 5‐year overall survival rate of only 8%, PDAC has now emerged as the fourth leading cause of cancer‐related deaths worldwide [1, 2]. The low overall survival rate is, in part, attributable to the late stage at diagnosis due to the absence of early specific symptoms. Additionally, the high propensity for metastasis and resistance to therapy substantially aggravate clinical outcome of PDAC [3, 4]. Tumor heterogeneity and cellular plasticity contribute to the biology of therapy resistance and ultimately to the poor overall prognosis of PDAC [5, 6]. In contrast, tumors exhibit few but frequently observed genetic alterations, which primarily include *KRAS*, *TP53*, *SMAD4*, and *CDKN2A* [7, 8]. The restricted number of genomic alterations is therefore unlikely to account for the diverse array of clinical disease phenotypes observed in PDAC patients.

Studying the pathobiology of the disease beyond genetic alterations has the potential to offer valuable insights into the molecular mechanisms that contribute to the diverse clinical phenotypes. Aiming for a patient stratification approach relevant to therapy and prediction of outcomes, PDAC was repeatedly characterized on the molecular level over the past decade [9, 10, 11, 12]. Despite variations in the reported number of molecular subgroups, substantial overlaps among the major molecular subtypes were identified [7, 13]. A recent consensus proposed a two‐factor prognostic subtype‐based classification based on distinct lineages: the less aggressive classical subtype and the basal‐like (or squamous) subtype [14]. These subtypes are primarily characterized by mutually exclusive gene expression patterns in gene sets associated with each subtype [11], hence implying a role of gene expression and dosage. With suggestions that patients with basal‐like tumors are less sensitive to first‐line treatments [15, 16, 17], molecular subtypes of PDAC may also have a future role in clinical decision‐making.

To integrate PDAC subtyping more seamlessly into clinical practice, targeted gene expression approaches such as NanoString profiling have been developed. Without the requirement for next‐generation sequencing, the process becomes more accessible for routine clinical use [18].

Proteomic panel approaches have also been suggested for PDAC classification. Notably, markers such as HNF1A and KRT81 have been identified for the classical or basal‐like subtype, respectively [19]. Other studies have proposed the utilization of the transcription factor *GATA6* for the stratification of the classical PDAC subtype [17]. Despite significant progress, targeted transcriptomic and proteomic approaches still rely on single‐lesion tumor biopsies. The identification of therapy‐induced tumor subtype switches [20] indicates the coexistence of both clinical phenotypes within the same tumor in a stimulus‐sensitive, highly dynamic cell continuum [15] and emphasizes the need for routine monitoring of PDAC subtypes and their dynamics. This is particularly relevant in light of clinical reports suggesting that resistance to FOLFIRINOX treatment is associated with the acquisition of the more resistant basal‐like subtype [17]. Unfortunately, the difficulty in obtaining tissue biopsies hinders their routine application for monitoring therapy‐induced phenotype switches. Moreover, tissue biopsies are limited by spatiotemporal bias, offering only a temporary snapshot of one part of a tumor. Consequently, they fail to capture the full extent of tumor heterogeneity and therapy‐induced tumor evolution.

Liquid biopsies, containing tumor‐relevant analytes, offer the potential to provide real‐time insights into the dynamic nature of the disease and its molecular architecture [21]. Among various liquid biopsy analytes, circulating cell‐free tumor DNA (ctDNA) has garnered significant attention and is recognized for its dual role in both predictive and prognostic assessments [22, 23, 24]. In contrast, other liquid biopsy analytes, like cell‐free RNA (cfRNA), have only recently gained attention. We and others have previously demonstrated that cfRNA holds both predictive and prognostic values. Additionally, it shows promise for early detection of tumor disease, tissue subtyping, and monitoring treatment response [25, 26, 27, 28, 29].

Analyzing liquid biopsies faces challenges due to background noise from sources irrelevant to the disease. Unlike ctDNA‐based approaches, where signal detection is hampered by germline DNA contamination, cfRNA has an advantage because it undergoes transcriptional amplification, enhancing the signal‐to‐noise ratio. This amplification allows for the straightforward detection of genes that are overexpressed in tumor tissue compared to healthy tissue. While the use of tissue‐based transcriptional data for PDAC subtype determination has been established, the use of cfRNA for PDAC subtyping to our knowledge has not been explored. Nevertheless, cfRNA has been reported to enable subtyping in lung cancer [29]. Given the advantages of cfRNA, it is plausible that cfRNA profiles could facilitate minimally invasive subtyping of PDAC as well.

In this proof‐of‐concept study, we investigated the clinical utility of cfRNA for minimally invasive PDAC subtyping. Employing whole transcriptome sequencing and RT‐ddPCR on patient plasma samples along with gene expression analysis of PDAC tissue samples, we identified several disease‐relevant cfRNA transcripts. Some of these transcripts exhibited subtype‐specific overexpression in both plasma and tumor samples, correlating with adverse outcomes and treatment failure. Additionally, the expression of some identified transcripts correlated with previously reported tissue‐based PDAC subtype markers.

Crucially, further profiling of these liquid‐based tumor subtype markers revealed increased abundance following FOLFIRINOX treatment. This observation aligns with previously reported therapy‐induced subtype switches after FOLFIRINOX treatment. Furthermore, the identified markers facilitated monitoring tumor recurrence post‐surgical resection. We characterized two of these cfRNA markers, *KDELC1* and *PTTG2*, using patient tissue and plasma samples as well as PDAC cell lines. *KDELC1*, a cfRNA transcript overexpressed in activated stromal cells of highly inflammatory tumors, was associated with poor overall survival, particularly in patients with basal‐like PDAC tumors. These findings underscore the potential clinical utility of cfRNA for minimally invasive PDAC subtyping, warranting further investigation.

## 2 Materials and methods

### 2.1 Patients and samples

Patient samples were retrospectively collected from various sources, including clinical trials and translational studies. Specifically, within the COMPASS trial (NCT02750657), plasma samples were serially collected from patients with localized disease both before and after surgical tumor removal. Additionally, baseline serum samples from advanced‐stage PDAC patients participating in the COMPASS trial were analyzed. It is noteworthy that all plasma/serum samples from the COMPASS trial originated from patients with well‐characterized tumors, and their molecular subtypes had been determined through transcriptomic analyses [16]. Gene expression data from the corresponding tumor samples were also reanalyzed. Secondly, serum samples were obtained from patients with locally advanced PDAC within the NEOLAP study (NCT02125136). The subtypes of these patients were not characterized, and they received treatment with either FOLFIRINOX (FFX) or gemcitabine plus nab‐paclitaxel (GnP). Plasma samples from patients with metastatic PDAC undergoing systemic chemotherapy with FOLFIRINOX were collected as part of local biobanking activities. Healthy blood donors were recruited from the Department of Transfusion Medicines at the University Hospital in Essen. All studies received approval from the corresponding local institutional ethics review boards, and all patients provided written informed consent for participation in the study.

### 2.2 Isolation of cfRNA

Cell‐free RNA was extracted from plasma and serum samples using the Plasma/Serum RNA Purification Mini Kit (cat # 55000, Norgen Biotek, Thorold, Canada) following the manufacturer's instructions with slight modifications. Briefly, samples were allowed to thaw at 4 °C overnight. 200 μL were transferred into a 2 mL safe‐lock Eppendorf tube and centrifuged at 16 000 ***g*** for 2 min. The supernatant was then transferred into a new 2 mL Eppendorf tube and lysed for 10 min at room temperature with three volumes of lysis buffer A supplemented with 0.01% beta‐mercaptoethanol. The lysate was precipitated with an equal volume of absolute ethanol and allowed to bind to the columns for 10 min at room temperature, followed by centrifugation at 3500 ***g*** for 2 min. The columns were incubated with RNase‐free DNase for 10 min at 37 °C and then washed four times with wash buffer A. Columns were dried, and cfRNA was eluted in 35 μL of elution buffer A. Cell‐free fRNA samples were quantified on a Quantus fluorometer (Promega, Walldorf, Germany) and stored at −80 °C. RNA isolated from cell lines was achieved by use of the SimplyRNA cells kit (cat #AS1390, Promega). All cell lines were authenticated by short tandem repeats (STR) analysis and were confirmed to be mycoplasma‐free. The commercially available cell lines used were: KP‐4 (RRID:CVCL_1338), PaTu 8988s (RRID:CVCL_1846), PaTu 8988t (RRID:CVCL_1847), HuP‐T4 (RRID:CVCL_1300), HPAF‐II (RRID:CVCL_0313), HPAC (RRID:CVCL_3517), MIA PaCa‐2 (RRID:CVCL_0428), PANC‐1 (RRID:CVCL_0480), PSN1 (RRID:CVCL_1644) were purchased from ATTC (Manassas, VA, USA) and patient‐derived primary PDAC cells PDC80, PDC85 and PDC62 were generated in‐house.

### 2.3 RNA sequencing and data analysis

RNA sequencing libraries were generated from cfRNA using the SMARTer Stranded Total RNASeq Kit v2 Pico Input Mammalian (Takara Bio USA, Inc, Kusatsu, Shiga, Japan) following the depletion of ribosomal RNA. The libraries were sequenced on a NovaSeq 6000 to ensure a minimum of 60 million 100 bp paired‐end reads. Following demultiplexing, read quality was assessed using FastQC, and low‐quality reads were trimmed with Trimmomatic. The sequencing reads were then mapped to the human reference genome version hg38, and read counting was conducted using STAR. A feature count matrix was created from the individual samples, and the measurement of differential transcript abundance was performed with edger [30]. Feature selection was performed using the boruta package [31]. Gene set enrichment analyses were conducted using the desktop version of the Gene Set Enrichment Analysis (GSEA) algorithm from the Broad Institute. Marker diagnostic performance was assessed using the combiroc package [32]. Tumor infiltration estimates were acquired using the online deconvolution tool timer [33].

### 2.4 cfRNA quantification by means of RT‐ddPCR

The abundance of *KDELC1* and *PTTG2* cfRNA transcripts was quantified using real‐time droplet digital PCR (RT‐ddPCR). Both of these genes have been previously linked to oncogenic pathways in human cancer and suggested as potential biomarkers [34, 35, 36, 37]. RT‐ddPCR was conducted using the 1‐Step RT‐ddPCR Advanced Kit for Probes (Bio‐Rad, Hercules, CA, USA) following the manufacturer's instructions. The reaction components were mixed to a final volume of 22 μL, and 20 μL were used for droplet generation in a QX100™/QX200™ droplet generator (Bio‐Rad). RT‐ddPCR reactions were performed in a C1000 Touch™ thermocycler (Bio‐Rad), and droplets were read in a QX100™/QX200™ droplet reader (Bio‐Rad). The raw transcript concentration (copies per 20 μL reaction) was used to determine the absolute transcript load per milliliter of plasma/serum using the appropriate formula:Copies/ml = C × EV × 1000/ISV × TPV

$$\text{Copies}\cdot {\mathrm{mL}}^{-1}=C\times \mathrm{EV}\times 1000/\mathrm{ISV}\times \mathrm{TPV}$$

with C, copies per 20 μL reaction; EV, elution volume; ISV, cfRNA input volume; TPV, total plasma/serum volume used of cfRNA isolation.

### 2.5 Quantitative mass spectrometry

Proteins (30 μg) were separated by SDS/PAGE, running for 4 cm and then divided into 8 pieces (each 0.5 cm in height). These pieces were subjected to trypsin digestion using a DigestPro MSi robotic system (INTAVIS Bioanalytical Instruments AG, Kölln‐reisiek, Schleswig‐Holstein, Germany), following previously established protocols [38]. LC–MS/MS analysis was conducted on an Ultimate 3000 UPLC system (Thermo Fisher Scientific, Waltham, MA, USA) directly coupled to an Orbitrap Exploris 480 mass spectrometer for a total duration of 60 min. Peptides were online desalted on a trapping cartridge (Acclaim PepMap300 C18, 5 μm, 300 Å wide pore; Thermo Fisher Scientific) for 3 min using a 30 μL·min^−1^ flow of 0.05% TFA in water. The analytical multistep gradient (300 nL·min^−1^) was performed using a nanoEase MZ Peptide analytical column (300 Å, 1.7 μm, 75 μm × 200 mm, Waters, Mainz, Germany) with solvent A (0.1% formic acid in water) and solvent B (0.1% formic acid in acetonitrile). Over 102 min, the concentration of B was linearly increased from 5% to 30%, followed by a rapid increase to 78%. After 2 min, the concentration of B was reduced to 2%, and a 10‐min equilibration step was added. Eluting peptides were analyzed in the mass spectrometer using data‐dependent acquisition (DDA) mode. A full scan at 60 000× resolution (380–1400 m/z, 300% AGC target, 45 ms maxIT) was followed by up to 1.5 s of MS/MS scans. Peptide features were isolated with a window of 1.4 m/z, fragmented using 26% NCE. Fragment spectra were recorded at 15 000× resolution (100% AGC target, 54 ms maxIT). Unassigned and singly charged eluting features were excluded from fragmentation, and dynamic exclusion was set to 20 s.

Data analysis was conducted using maxquant (version 1.6.14.0) [39], employing an organism‐specific database extracted from Uniprot.org under default settings (human containing 74 811 entries as of 27.02.2020). False discovery rate (FDR) cut‐off values were set at 0.01 for both the peptide and the protein levels. The match between runs (MBR) option was enabled to transfer peptide identifications across RAW files based on accurate retention time and m/z. Label‐free quantification (LFQ) was performed using the MaxLFQ algorithm [40], with a minimum of two quantified peptides per protein = required for protein quantification. Following the Perseus recommendations [41], protein groups with valid LFQ values in 70% of the samples from at least one of the conditions were used for statistical analysis. Missing LFQ values were imputed with random values drawn from a downshifted (1.8 standard deviation) and narrowed (0.3 standard deviation) intensity distribution of the individual samples. Statistical analysis for LFQ values was carried out using the r‐package “limma” [42]. The *P*‐values were adjusted using the Benjamini–Hochberg method to account for multiple testing [43]. (These methods have been described in a similar manner in previous works.).

### 2.6 Multiplexed immunofluorescent (IF) histological staining

Multiplexed immunofluorescence (IF) was carried out using the Opal multiplex system (Akoya Biosciences, Marlborough, MA, USA) following the manufacturer's instructions. Briefly, formalin‐fixed paraffin‐embedded (FFPE) sections were deparaffinized, fixed with 4% paraformaldehyde, and subjected to antigen retrieval using tris/EDTA (pH 9) for heat‐induced epitope retrieval. Each section underwent multiple rounds of staining, involving endogenous peroxidase blocking, protein blocking, primary antibodies (PanCK, Abcam, ab6401 (Cambridge, UK); HPA043946, Sigma‐Aldrich, St. Louis, MO, USA), and the corresponding secondary horseradish peroxidase‐conjugated polymer (Zytomed Systems, Berlin, Germany or Akoya Biosciences). Tyramide signal amplification was employed to bind each horseradish peroxidase‐conjugated polymer with different fluorophores. To remove antibodies before the next round of staining, additional antigen retrieval in heated Tris/EDTA (pH 9) was performed. Following all sequential staining reactions, sections were counterstained with DAPI (Vector Lab, Newark, CA, USA). Using a Zeiss Axio Scanner Z.1 (Carl Zeiss AG, Oberkochen, Germany) at 10× objective magnification, slides were scanned and digitalized. Quantification of individual and/or co‐expressing markers in the multiplexed immunofluorescence images was performed using halo software (Indica Labs, Albuquerque, NM, USA).

### 2.7 KRT77 ELISA assay

Quantification of *KRT77* in serum samples was conducted using the Keratin, type 2 cytoskeletal 1b (KRT77) ELISA Kit (cat# MBS1606438, My Biosource, San Diego, CA, USA) following the manufacturer's instructions. Briefly, after allowing the reagents to equilibrate at room temperature for 30 min, 40 μL of serum from each patient were added to duplicate wells of the *KRT77* antibody pre‐coated ELISA plate. 10 μL of *KRT77* antibody were then added to form a sandwich, followed by 50 μL of streptavidin‐conjugated horseradish peroxidase. The samples were incubated at 37 °C for 60 min. The plate was washed five times for 30 s each with 300 μL of wash buffer. Subsequently, samples were incubated with 50 μL each of substrate solution A and substrate solution B for 10 min at room temperature in the dark. After adding 50 μL of stop solution, the optical density was determined at 450 nm.

### 2.8 Statistical analyses

Overall survival was calculated from the time of enrollment until death or withdrawal. Samples were categorized as showing high gene expression if their expression was above an optimized cut‐off expression value in the dataset; otherwise, samples were considered to have low gene expression. All analyses were conducted in the R environment with R version 4.2.0 or GraphPad Prism version 8.0 (GraphPad Software, Inc, La Jolla, CA, USA). Statistical significance was set to a *P*‐value < 0.05, and non‐parametric tests were employed when the data distribution did not follow a Gaussian distribution or when the dataset was too small to assume any normal distribution. For overall survival analyses, patients were dichotomized based on the expression of the candidate genes. The dichotomization cut‐off value was determined using the surv_cutpoint function of the R survminer package, and the survival package was used for computing overall survival estimates. Correlation analysis for pairs of genes was performed with the corr package, and the CombiROC package was utilized to determine biomarker diagnostic performance.

## 3 Results

### 3.1 Patient cohort description

In this study, we analyzed over 600 tissue and liquid samples, which were collected from various sources, including the COMPASS trial (NCT02750657), the NEOLAP trial (NCT 02210559), institutional translational studies at the University Hospital Essen (referred to as the UK‐Essen cohort), and the Department of General, Visceral and Transplantation Surgery, University of Heidelberg (HD cohort). From the COMPASS trial, 34 pre‐ and post‐surgery plasma samples from 14 patients (4 basal‐like and 10 classical) with localized disease were included. Additionally, serum samples from 20 metastatic PDAC patients (10 classical and 10 basal‐like) enrolled within the COMPASS trial were analyzed. Tumor gene expression data for 253 patients (with transcriptome‐derived molecular subtypes) from the COMPASS trial were available via a data transfer agreement. Either these patients were treated with FOLFIRINOX or with gemcitabine plus nab‐paclitaxel and response data were available for all patients included in the analyses. Of this number, 33 cases were patients with localized tumors. From the NEOLAP cohort, pre‐surgery serum samples from 122 locally advanced PDAC patients were analyzed. The UK‐Essen cohort comprised 24 plasma samples from 12 patients (baseline and after FOLFIRINOX treatment) with metastatic disease. Additional gene expression data from more than 190 localized PDAC patients, 59 chronic pancreatitis, and 41 normal pancreas tissues were reanalyzed (HD cohort). Lastly, 24 plasma and 24 serum samples from healthy blood donors obtained from the Department of Transfusion Medicines at the University Hospital Essen were included in the analysis. The study was approved by the ethics committee at the medical faculty of the University of Duisburg‐Essen (17‐7729‐BO), and all studies were performed in accordance with the standards set by the Declaration of Helsinki. The sampling time lines of the different clinical trials can be obtained under the provided registration numbers. All other samples were collected at the outpatient unit of the West German Cancer center of the University Hospital Essen between January 2018 and December 2021.

### 3.2 cfRNA sequencing reveals reliable tumor subtype markers

**Fig. 1:**
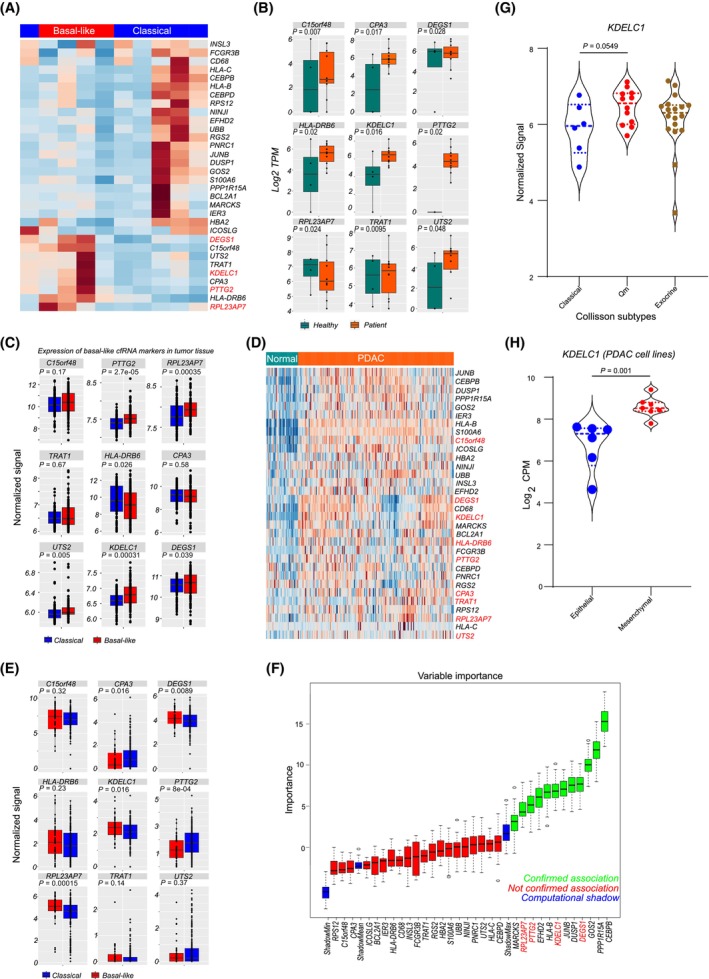
Whole transcriptome sequencing of circulating cell‐free RNA reveals reliable disease‐relevant tumor subtype markers. (A) Heatmap of differentially abundant cfRNA (cell‐free RNA) transcript in plasma samples from PDAC (Pancreatic ductal adenocarcinoma) patients (6 classical and 4 basal‐like) with localized tumors from the COMPASS trial. Only coding transcripts showing overexpression in PDAC tumors (HD cohort) are presented. Genes highlighted in red are further investigated in this study. (B) Boxplot of selected cfRNA‐defined basal‐like subtype marker in plasma samples from PDAC patients (*N* = 10) and healthy blood donors (*N* = 4). Data were generated by whole transcriptome cfRNA sequencing. *P*‐values were calculated with a Mann–Whitney *U*‐test. (C) Boxplot of cfRNA‐defined basal‐like subtype markers in gene expression data from PDAC tumors of basal‐like and classical subtype in the HD cohort (*N* > 190). *P*‐values are obtained from a Wilcoxon test. (D) Heatmap of differentially abundant cfRNA transcripts in PDAC tumors tissue (*N* > 190) and non‐tumor pancreas in the HD cohort (*N* > 40). cfRNA‐defined basal‐like subtype markers are indicated in red. (E) Boxplot of cfRNA‐defined basal‐like subtype markers in gene expression data from PDAC tumors of basal‐like and classical subtype in the COMPASS cohort (*N* > 250). *P*‐values are obtained from a Wilcoxon test. (F) Variable of importance boxplot of differentially abundant cfRNA transcripts and their association with the basal‐like subtype and classical PDAC tumor subtypes. (G) Violin plot showing the expression of a selected cfRNA‐defined basal‐like tumor subtype marker (*KDELC1*) in data for different PDAC tumor subtypes from the Collisson study (Qm, overlapping with Moffitt's basal‐like subtype). Qm here refers to the Quasi‐mesenchymal subtype of pancreatic ductal adenocarcinoma (PDAC). *P*‐values are estimated with an ANOVA test. (H) Violin plot presenting the expression of *KDELC1* in six epithelial and six mesenchymal PDAC cell lines. Presented *P*‐values are calculated with a Mann–Whitney *U*‐test. All boxplots are presented with the full dataset and the mean is indicated within the boxplot together with the minimum and maximum data points.

In an exploratory approach, whole circulating cell‐free RNA (cfRNA) sequencing was conducted on 10 plasma‐derived cfRNA samples (4 basal‐like and 6 classical) from patients with localized disease within the COMPASS trial, where the tumor molecular subtypes were transcriptionally determined. Coding transcripts with log2‐fold change ≥ 1 and false discovery rate (FDR) < 0.05, which were differentially abundant between basal‐like and classical cfRNA samples and overexpressed in patient tumor samples compared to non‐tumor pancreas, were selected (Fig. 1A). A total of 32 differentially abundant coding transcripts were identified, with nine of them abundant in cfRNA from basal‐like samples. The profiles of these nine basal‐like associated markers in cfRNA from patient and healthy plasma samples were then investigated, revealing that most of the assessed cfRNA transcripts were more abundant in patient samples compared with healthy donor plasma (Fig. 1B).

Of the nine basal‐like‐associated cfRNA markers, five (*PTTG2*, *RPL23AP7*, *UTS2*, *KDELC1*, and *DEGS1*) were found to be overexpressed in basal‐like tumor tissue compared with tumors of the classical subtype within the HD tissue cohort (Fig. 1C). All cfRNA markers were analyzed for their disease relevance and were observed to be overexpressed in tumor tissue compared with non‐tumor pancreas (Fig. 1D). In an independent cohort of 253 tumor biopsies from the COMPASS trial, the association of the basal‐like cfRNA markers was reassessed. In this cohort, three of the basal‐like markers (*KDELC1*, *DEGS1*, and *RPL23AP7*) were significantly overexpressed in basal‐like tumors (Fig. 1E). Some genes, especially *PTTG2*, *CPA3* and *UTS2* showed a discrepant pattern in both cohorts, which might be attributed to differences in the cellular architecture associated with sampling, prior treatment or analytical platform discrepancies. Using a random forest‐based classifier, the Boruta package was employed to classify all 32 transcripts based on their association with the different subtypes and capture the important features associated with each subtype (Fig. 1F). In both liquid and tissue data, the transcripts *KDELC1*, *DEGS1*, and *RPL23AP7* consistently showed association with the basal‐like subtype. These transcripts, along with *PTTG2*, which was equally significant in our cohort and exhibited similar trends as the other selected markers, were the focus of our analysis. Utilizing data from the Collisson subtype study [9], we observed a higher but not significant expression of *KDELC1* in quasi‐mesenchymal (Qm, overlapping with Moffitt's basal‐like subtype) tumors compared with tumors of the classical subtype (Fig. 1G). Tumors of the exocrine subtype had comparable expression as tumors with the classical subtypes. Finally, we examined the expression of *KDELC1* in PDAC cell lines with epithelial (HPAC, HPAF‐II, Patu8988s, HupT‐4, PDC4 and PDC62, the PDC cells are primary patient‐derived cell lines) and mesenchymal phenotypes (Patu8988t, PSN1, MiaPaca‐2, Panc‐1, KP4, PDC80, PDC85), and significant overexpression was observed in the mesenchymal cell lines (Fig. 1H). We further evaluated the expression of *KDELC1* and *PTTG2* in samples from non‐tumor pancreas, pancreatitis and PDAC tissues from the HD cohort and observed significantly higher expression in PDAC (Fig. S1). Collectively, these findings provide support for the use of circulating cell‐free RNA (cfRNA) in identifying disease‐relevant minimally invasive tumor subtype markers.

### 3.3 cfRNA‐defined basal‐like markers are associated with adverse outcome

**Fig. 2:**
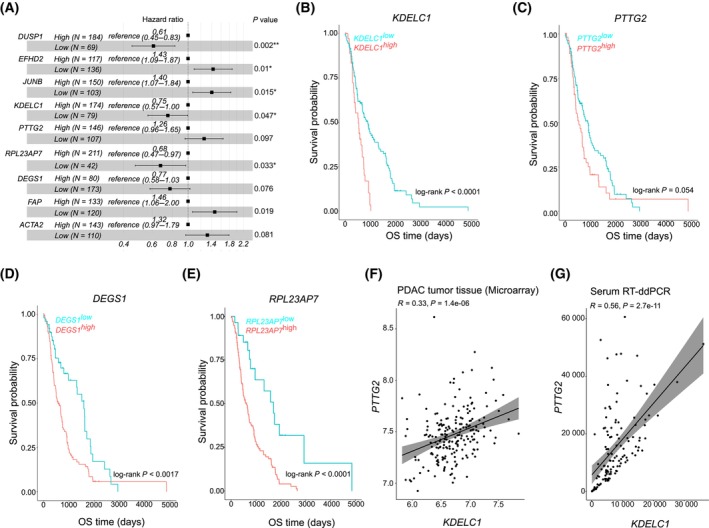
cfRNA‐defined tumor subtype markers have prognostic value. (A) Forest plots of multivariate Cox proportional hazards analysis of cfRNA‐defined tumor subtype marker in tumor gene expression data from the COMPASS trial (*N* > 250). The bars represent the 95% confidence intervals. *P*‐values are estimated by a log‐rank test. (B–E) Kaplan–Meier overall survival curves for randomly selected basal‐like subtype markers (*KDELC1*, *PTTG2*, *DEGS1* and *RPL23A7*) in the HD (Heidelberg) cohort (*N* > 160). (F) Correlation scatter plot showing the correlation between two cfRNA‐defined basal‐like subtype markers in tumor samples within the HD cohort. (G) Scatter plot showing the correlation between two cfRNA‐defined basal‐like subtype markers in cfRNA samples from the NEOLAP trial (*N* > 120). All boxplots are presented with the full dataset and the mean is indicated within the boxplot together with the minimum and maximum data points. Except otherwise stated, the p‐values are calculated by a *t*‐test. In figures (F) and (G), the *x*‐axis represents the expression of *KDELC1* and the *y*‐axis represents *PTTG2* expression.

Previous subtyping studies have indicated the association of basal‐like tumors with poor overall survival and treatment failure [9, 10, 11, 12, 44]. Therefore, we investigated if our circulating cfRNA‐defined basal‐like subtype markers were associated with poor outcomes. Using data from the COMPASS trial, we observed that low expression of the cfRNA basal‐like markers *KDELC1* (HR = 0.75, 95% CI 0.57–1.00, *P* = 0.047), *RPL23AP7* (HR = 0.68, 95% CI 0.47–0.97, *P* = 0.033), and *DEGS1* (HR = 0.77, 95% CI 0.58–1.03, *P* = 0.076) was associated with better overall survival. Among the classical markers, low expression of *EFHD2* (HR = 1.43, 95% CI 1.09–1.87, *P* = 0.01) and *JUNB* (HR = 1.40, 95% CI 1.07–1.84, *P* = 0.015) was associated with worse overall survival, all in a multivariate analysis (Fig. 2A). These findings were partially recapitulated in data from the HD tumor tissue cohort, where overexpression of *KDELC1*, *RPL23A7*, and *DEGS1*, but not *PTTG2*, was associated with worse overall survival (Fig. 2B–E, respectively). A correlation analysis between *KDELC1* and *PTTG2* revealed a significant positive correlation between the expression of both transcripts in tumor tissue (Fig. 2F, HD cohort) and serum‐derived cfRNA (Fig. 2G, NEOLAP cohort).

**Fig. 3:**
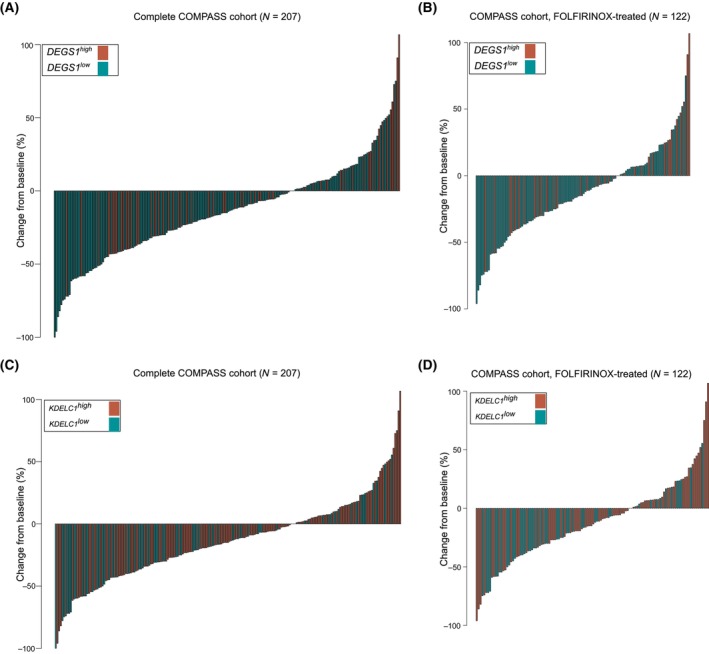
Overrepresentation of cfRNA‐defined basal‐like tumor subtype markers is associated with systemic chemotherapy response. Waterfall plots show the distribution of the best overall response to treatments in patients within the COMPASS cohort. Patients are dichotomized to either *DEGS1* ^*high*^ and *DEGS1* ^*low*^ (A, B) or *KDELC1* ^*high*^ and *KDELC1* ^*low*^ expression tumor tissue (C, D), respectively. Dichotomization is based on a predetermined cut‐off value defined in the Cox proportional hazards analysis. (A, C) Treatment with FOLFIRINOX and gemcitabine plus nab‐paclitaxel (GnP); (B, D) treatment with FOLFIRINOX.

Next, patient treatment response data from the COMPASS cohort (*N* = 253) were used to investigate the association between the cfRNA‐defined subtype markers and therapy response. Overexpression of *DEGS1* was associated with a poor response to systemic treatment with FOLFIRINOX (FFX) and gemcitabine plus nab‐paclitaxel (GnP) (Fig. 3A). In FFX‐treated patients, low expression of *DEGS1* was more likely to predict treatment response compared with GnP treatment (Fig. 3B, Fig. S2A). Similarly, low expression of *KDELC1* was associated with a better response to systemic treatment with FFX or GnP (Fig. 3C). As with *DEGS1*, low expression of *KDELC1* was more likely to predict treatment response in FFX‐treated patients than in GnP‐treated patients (Fig. 3D, Fig. S2B). These data are consistent with previous findings associating basal‐like tumors with treatment failure and poor outcomes.

### 3.4 cfRNA transcript abundance of *KDELC1* is predictive

**Fig. 4:**
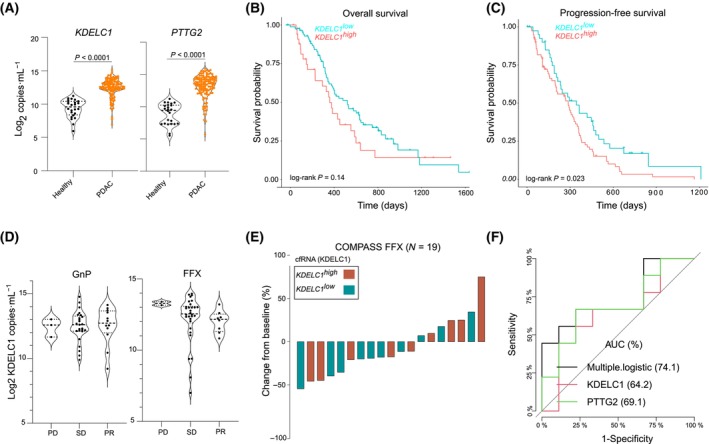
cfRNA abundance of *KDELC1* predicts treatment response. (A) Violin plot for the cfRNA abundance of *KDELC1* (left panel) and *PTTG2* (right panel) in serum samples from the NEOLAP study (*N* > 120) and healthy blood donors (*N* = 24). cfRNA transcript quantification was achieved by means of RT‐ddPCR using Fam‐ and Hex‐labeled probes. *P*‐values are calculates by means of a Wilcoxon test. (B) Kaplan–Meier overall survival (OS) curve for patients with high and low abundance of *KDELC1* cfRNA transcript in serum samples from the NEOLAP cohort. (C) A Kaplan–Meier progression‐free survival curve for patients with high and low abundance of *KDELC1* cfRNA transcript in serum samples from the NEOLAP cohort. (D) Violin plots showing the *KDELC1* cfRNA abundance in serum from patients in Arm A (GnP: gemcitabine plus nab‐paclitaxel; left panel) and Arm B (GnP and FFX, right panel) of the NEOLAP trial. Patients were classified as PD, SD, or PR based on RECIST 1.1 (PD, progressive disease; PR, partial response; SD, stable disease). (E) A waterfall plot showing the distribution of best overall response in patients treated with FOLFIRINOX and GnP within the COMPASS cohort. Patients are dichotomized to *KDELC1* ^*high*^ and *KDELC1* ^*low*^ cfRNA abundance in serum samples. Dichotomization is based on a predetermined cut‐off defined using the surminer package. (F) A receiver operator characteristic curve for the performance of *KDELC1* and *PTTG2* cfRNA transcript for the identification of basal‐like and classical tumors in 20 metastatic PDAC patients from the COMPASS trial.

We next investigated whether *KDELC1* cfRNA transcript abundance could predict patient outcomes. We focused on *KDELC1* for several reasons: (a) It had been reported to be associated with several cancers and is therefore likely associated with neoplastic transformation; (b) it showed a very clear difference in expression between non‐tumor and PDAC tumor tissue (Fig. 1D), and (c) a reliable probe design for the quantification of its transcript in plasma. To this end, we analyzed the cfRNA abundance of *KDELC1* and *PTTG2* in 122 serum samples from patients with locally advanced PDAC from the NEOLAP trial. Serum abundance of both transcripts was observed in PDAC patients compared with healthy blood donors (Fig. 4A). Among PDAC patients, higher levels of *KDELC1* cfRNA transcript were associated with worse progression‐free survival (Fig. 4C). Moreover, patients treated with FFX within the NEOLAP trial were more likely to respond if their *KDELC1* cfRNA abundance was lower. Such a trend was not observed in GnP‐treated patients within the same cohort (Fig. 4D).

Subsequently, the evaluation of tumor response to treatment in 19/20 patients from the COMPASS trial was performed. As depicted in Fig. 4E, 7/12 patients with a measurable decrease in tumor volume had lower serum levels of *KDELC1* cfRNA transcript as opposed to 3/7 who showed tumor progression. Analysis of *KDELC1* and *PTTG2* cfRNA abundance in 20 serum samples from metastatic patients with transcriptome‐defined tumor subtype from the COMPASS trial showed that the AUC for *KDELC1* and *PTTG2* was 0.64 and 0.69, respectively (Fig. 4F). Taken together, these findings suggest that *KDELC1* cfRNA transcript abundance has some predictive value.

### 3.5 Multi‐omic liquid biopsy analytes have stronger predictive value

**Fig. 5:**
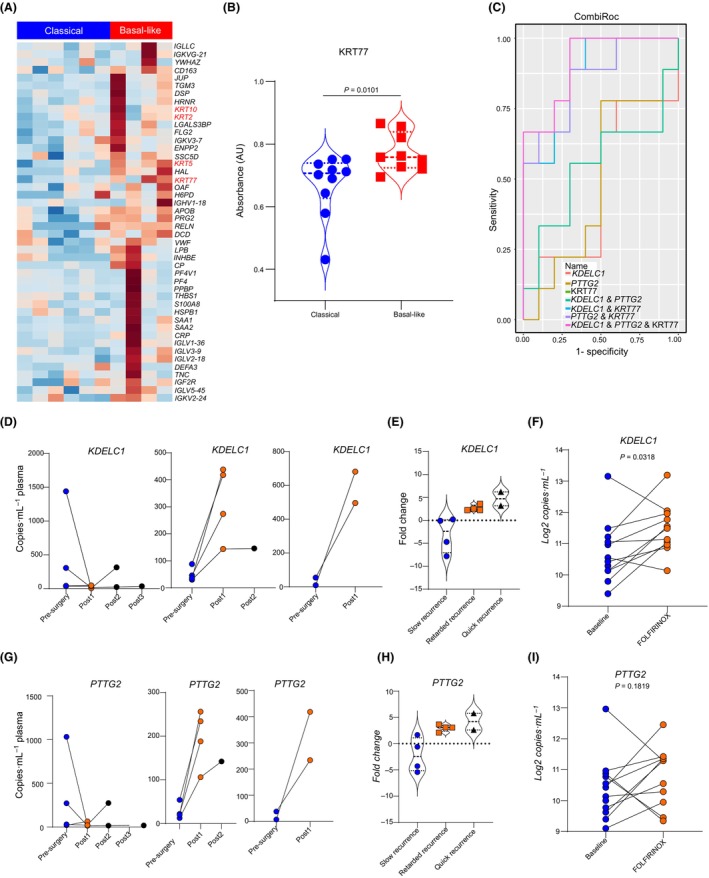
Multi‐omic liquid biopsy analysis improved biomarker performance. (A) A heat map showing differential protein abundance in plasma samples from classical and basal‐like PDAC tumors. Protein abundance was analyzed by label‐free quantitative mass spectrometry. All keratin proteins are highlighted in red. (B) A violin plot presenting the expression of KRT77 in serum samples from patients with classical and basal‐like PDAC tumors. (C) A combined receiver operator characteristic curve (ROC) showing the diagnostic performances of different subtype markers as single markers and in combinations. ROC curves were generated using the combiroc package. (D) Line plots showing the expression profile of *KDELC1* in plasma samples collected before surgical tumor resection and at different time points after surgery for patients who showed a slow tumor recurrence (left panel, *n* = 4), retarded tumors recurrence (middle panel, *n* = 4), and fast tumor recurrence (right panel, *n* = 2). (E) A violin plot showing *KDELC1* cfRNA transcript fold change in patients with slow, retarded, and fast recurrence. (F) Line plots showing the cfRNA abundance profile of *KDELC1* in patient plasma samples collected before and after once cycle of FOLFIRINOX (FFX) treatment. (G) Line plots showing the expression profile of *PTTG2* in plasma samples collected before surgical tumor resection and at different time points after surgery for patients who showed a slow tumor recurrence (left panel, *n* = 4), retarded tumors recurrence (middle panel, *n* = 4), and fast tumor recurrence (right panel, *n* = 2). (H) A violin plot showing *PTTG2* cfRNA transcript fold change in patients with slow, retarded, and fast recurrence. (I) Line plots showing the cfRNA abundance profile of *PTTG2* in patient plasma samples collected before and after once cycle of FFX (FOLFIRINOX) treatment. The violin plots within this figure shows the complete dataset with the mean as well as the minimum and maximum data points. Except otherwise stated, all presented *P*‐values are estimated by a Mann–Whitney *U*‐test.

In order to enhance the strength of the liquid biopsy cfRNA subtype markers, quantitative protein mass spectrometry on the same plasma samples as those that were analyzed by whole transcriptome sequencing (10 samples: 4 basal‐like and 6 classical) was undertaken. Among the differentially abundant proteins, several keratins, including *KRT2*, *KRT5*, *KRT10*, and *KRT77*, were overrepresented in plasma samples from patients with basal‐like tumors (Fig. 5A). Using enzyme‐linked immunosorbent assay (ELISA), we analyzed an additional 20 samples from the COMPASS metastatic cases, and indeed could show that KRT77 was overrepresented in serum samples from patients with basal‐like tumors (Fig. 5B). We then evaluated the performance of the single and different combinations for the individual cfRNA and protein markers. In this analysis, we investigated if the individual cfRNA transcript (*KDELC1* and *PTTG2*) or the proteomic marker (*KRT77*) would alone allow for unambiguous determination of tumors subtypes of a combination of some or all markers was better (*N* = 20). Individually, both the cfRNA and proteomic markers did not show any meaningful performance. Combination of at least two markers (irrespective of analyte type) substantially improved the performance, meanwhile combination of all three markers performed slightly better than two markers (Fig. 5C).

### 3.6 cfRNA transcript abundance predicts disease relapse

To better understand a clinical relevance of our markers, we investigated if the identified markers allow for monitoring disease dynamics. To this end, we analyzed the cfRNA transcript profile of *KDELC1* and *PTTG2* in serially collected plasma samples from patients who underwent surgical resection (COMPASS cohort). Respective data were available for 10 cases. Of the 10 patients, four showed very slow tumor recurrence with the tumor recurring beyond a year after resection, four patients had a retarded recurrence which occurred within 6–12 months after resection, and two showed a rapid recurrence, which occurred within less than 6 months after the initial resection. Serial monitoring of the *KDELC1* cfRNA transcript from all 10 patients revealed that patients showing slow tumor recurrence had a drop in the cfRNA transcript levels of the gene after surgical resection (Fig. 5D). In patients with retarded and quick recurrence, the cfRNA transcript levels of *KDELC1* increased sharply after surgery and remained high. Comparing the *KDELC1* cfRNA fold change between post‐ and pre‐surgery time points, patients with quick recurrence had a significantly higher positive change followed by those with retarded recurrence (Fig. 5E). We further analyzed the cfRNA transcript profile of *KDELC1* in metastatic PDAC patients receiving FFX systemic therapy at the pre‐treatment time point and after the first treatment cycle and observed a significant increase in the *KDELC1* cfRNA transcript profile in the post‐treatment samples (Fig. 5F). Similar findings were observed for *PTTG2* cfRNA transcript dynamics (Fig. 5G–I).

### 3.7 *KDELC1* overexpression is associated with activated stroma and mesenchymal genes

**Fig. 6:**
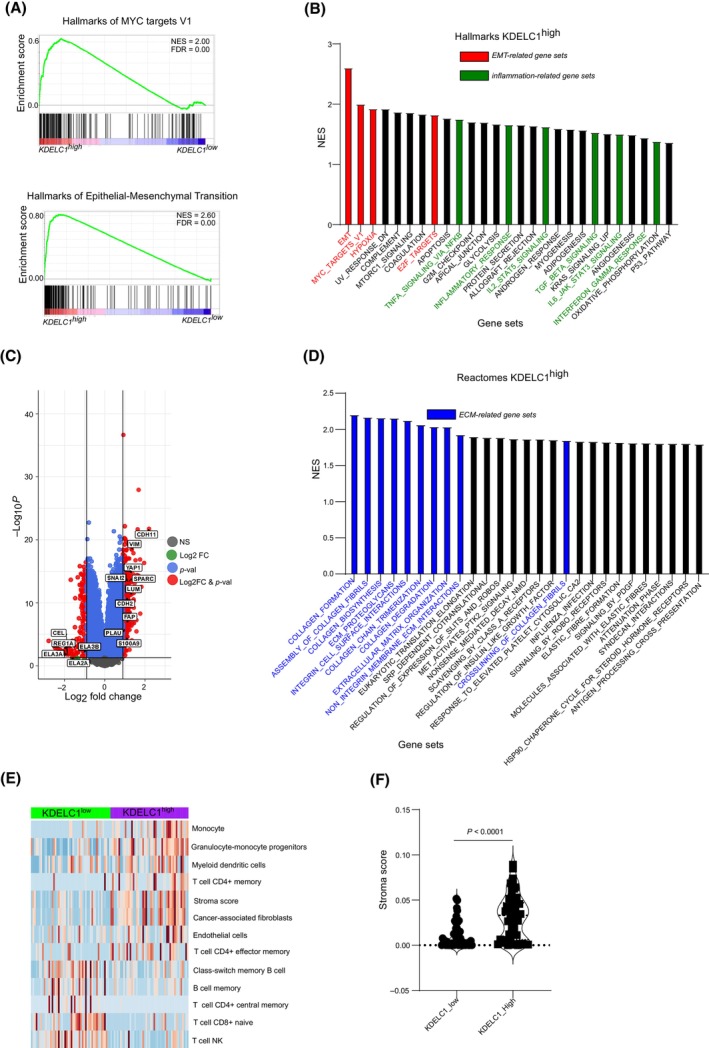
Overexpression of KDELC1 is associated with EMT‐related process and extracellular matrix reorganization. (A) Gene set enrichment plot showing the enrichment of the hallmarks of *MYC* targets (upper panel) and the hallmarks of epithelial‐mesenchymal transition in tumor samples showing high expression of *KDELC1*. (B) A bar plot showing hallmark gene sets with significant enrichment in tumor samples with high *KDELC1* expression. Gene sets were considered to be significantly enriched if the false discovery rate was *P* < 0.05 and the normalized enrichment score above 1.5. The bar height represents the normalized enrichment score (NES), EMT refers to epithelial‐to‐mesenchymal transition (EMT). (C) Volcano plot showing differentially expressed genes in *KDELC1* ^high^ and *KDELC1* ^low^ tumor samples. (D) A bar plot showing reactome gene sets with significant enrichment in tumor samples with high *KDELC1* expression. Gene sets were considered to be significantly enriched if the false discovery rate was *P* < 0.05 and the normalized enrichment score above 1.5. (E) Heat map showing tumor infiltration estimates of different stroma cells in samples with high and low *KDELC1* expression. Tumor infiltration estimation was performed using the only deconvolution tool TIMER. Data are only presented for cell types with at least a 0.001% infiltration observed in at least 10% of samples. (F) Violin plot presenting the stroma score for tumors with high and low expression of KDELC1. Presented *P*‐values are derived from a Wilcoxon test.

**Fig. 7:**
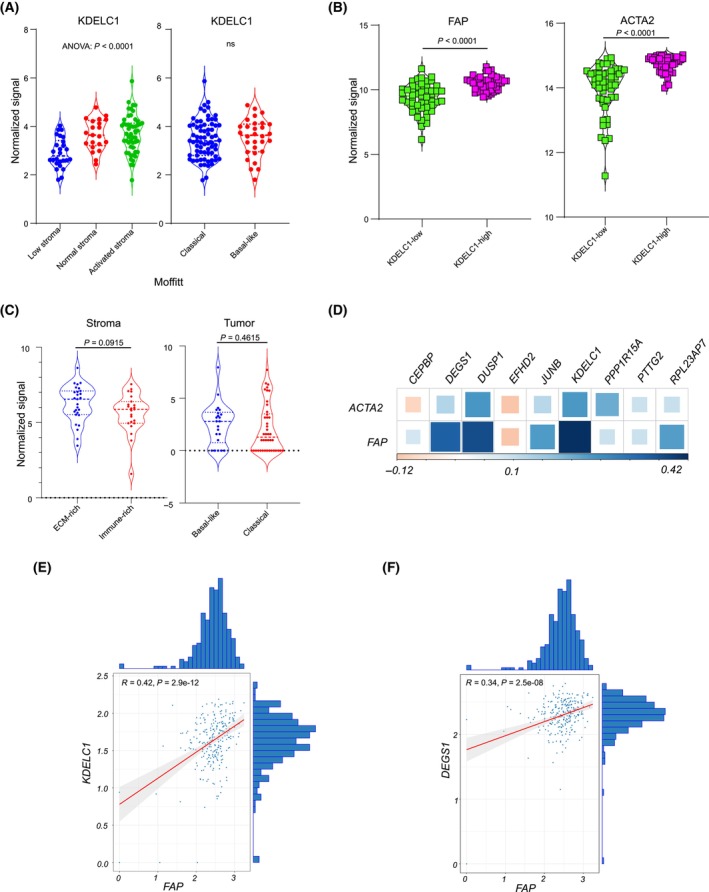
*KDELC1* overexpression is associated with activated stroma and mesenchymal genes. (A) Violin plots showing the expression of *KDELC1* in different stroma subtypes (left panel) and tumor subtypes (right panel) for the PDAC subtyping study of Moffit et al. [11]. For the purpose of this study, data from the Moffitt study were only included for (pancreatic ductal adenocarcinoma) PDAC tumors (primary and metastatic). (B) Violin plots for the expression of *FAP* (left panel) and *ACTA2* (right panel) in PDAC tumors samples within the HD cohort with high and low *KDELC1* expression. Data from the HD cohort are bulk microarray‐based transcriptome analysis. (C) Violin plot for the expression of *KDELC1* in the stroma (left panel) and epithelial tumor compartment (right panel) in laser microdissected PDAC samples from the Mauer et al. [44], PDAC subtyping study. ECM here refers to the extracellular matrix. *P*‐values for figures B and C are derived from a Wilcoxon test. All other *P*‐values within this figure are derived from a *t*‐test. (D) A correlation heatmap between cfRNA‐defined tumor subtype marker and stromal markers in the COMPASS metastatic cohort (*N* > 250). (E) A correlation scatter plot for the correlation between *KDELC1* and *FAP* in the COMPASS cohort. The *x*‐axis represents the expression of FAP and the *y*‐axis represents *KDELC1* expression. (F) A correlation scatter plot for the correlation between *DEGS1* and FAP in the COMPASS cohort. The violin plots within this figure shows the complete dataset with the mean as well as the minimum and maximum data points. The *x*‐axis represents the expression of *FAP* and the *y*‐axis represents *DEGS1* expression.

Lastly, we analyzed pathways associated with high *KDELC1* expression. To this end, we dichotomized the HD cohort data set into KDELC1^high^ and *KDELC1* ^*low*^ expression (based on a predetermined cut‐off value used for the Kaplan–Meier analyses). Gene set enrichment analysis revealed significant enrichment in the hallmarks of epithelial‐mesenchymal transition in *KDELC1* ^*high*^ samples as well as enrichment in the hallmarks of MYC targets (Fig. 6A,B), both of which are associated with basal‐like tumors [45]. Differential gene expression analysis comparing *KDELC1* ^*high*^ and *KDELC1* ^*low*^ samples revealed overexpression of mesenchymal markers such as *SNAI2*, *VIM*, and *CDH2* in *KDELC1* ^*high*^ samples. Similarly, markers of activated stroma such as *FAP*, *LUM*, and *SPARC* were overexpressed in *KDELC1* ^*high*^ samples (Fig. 6C). The overexpression of stromal markers in the *KDELC1* ^*high*^ samples prompted us to perform further analyses. Reactome pathway analysis revealed enrichment in several pathways associated with the extracellular matrix (ECM) (Fig. 6D). Deconvolution of the HD cohort data revealed that *KDELC1* ^*high*^ samples had a higher stroma score compared with *KDELC1* ^*low*^ samples (Fig. 6E,F). These findings were subsequently validated by multiplex immunofluorescence staining of a tissue microarray of more than 140 samples (Fig. S3). Wondering if *KDELC1* is a subtype marker associated with the ECM as previously described [44], a reanalysis of gene expression from Moffitt et al. [11] was undertaken and revealed that *KDELC1* was overexpressed in tumors with activated stroma with no obvious difference observed in the tumor compartment (Fig. 7A). To validate these findings, we analyzed the expression of *FAP* and *ACTA2* in *KDELC1* ^*high*^ and *KDELC1* ^*low*^ samples and observed higher expression of both genes in KDELC1^*high*^ samples (Fig. 7B). We next used data from laser microdissected PDAC tumors from Mauer et al. [44] to interrogate *KDELC1* expression in the different tumor compartments. ECM‐rich tumors (which are associated with basal‐like features) had higher expression of *KDELC1* compared with immune‐rich tumors (which are associated with classical tumors). As with the Moffitt data, no difference was observed in the epithelial compartment of the tumor (Fig. 7C). We then performed a correlation analysis between *ACTA2* and *FAP* with the cfRNA‐defined subtype markers in tumor gene expression data from the COMPASS trial. *FAP* showed the strongest correlation with *KDELC1*, *DEGS1*, and *DUSP1*. A similar but weaker pattern was observed for *ACTA2* (Fig. 7D–F). Together, these findings suggest that *KDELC1* is an ECM‐rich tumor subtype marker, associated with activated stroma.

## 4 Discussion

We here demonstrate a proof‐of‐concept investigation into minimally invasive liquid biopsy‐based biomarkers for determining tumor subtypes and monitoring disease recurrence in pancreatic ductal adenocarcinoma (PDAC). Whole transcriptome cfRNA analysis, coupled with label‐free quantitative protein mass spectrometry and RT‐ddPCR, was performed on samples with well‐characterized tumor subtypes. We identified 32 disease‐related subtype‐specific cfRNA transcripts. Most of these genes have already been reported to be associated with various oncogenic processes, such as *JUNB* regulating TGFß signaling, invasion, and metastasis [46, 47]. *PTTG2* has been implicated in glioblastoma tumorigenesis as well as in head and neck squamous cell carcinoma [36, 48], *UTS2* has been reported to possess prognostic value in colorectal and breast cancers [49, 50], *TRAT1* in early breast cancer prognosis [51] and *DUSP1* in sarcoma progression [35, 52]. *KDELC1* is involved in the regulation of NOTCH signaling and is overexpressed in several cancers including PDAC. Loss of *KDELC1* function leads to delay in tumor formation [35, 53]. Notch signaling is required for the progression of IPMNs into PDAC [54]. *DEGS1* is an intermediate enzyme in the synthesis of ceramide at the endoplasmic reticulum and the plasma membrane via the de novo pathway, and induces apoptosis in pancreatic cancer cells [55]. Of the 32 identified genes, nine were found to be associated with basal‐like tumors. Through feature prioritization, four cfRNA transcripts (*KDELC1*, *PTTG2*, *DEGS1*, and *RPL23AP7*) were selected as biomarkers for basal‐like tumors. Following a literature search, three of these transcripts (*PTTG2*, *DEGS1*, and KDELC1) were chosen for additional validation. Notably, *PTTG2* had already been reported to be associated with the aggressiveness of several cancer entities [37, 48, 56]. *KDELC1* is involved in Notch signaling, a known cancer‐associated signaling pathway [35]. We further analyzed these two transcripts to explore their potential roles in disease detection and subtype determination in liquid biopsies. Additionally, *DEGS1* was subjected to further analysis for predicting treatment response, along with *KDELC1*. The cfRNA transcript profiles of both *KDELC1* and *PTTG2* could successfully predict tumor relapse after surgical resection. In fact, cfRNA transcript profiles have been previously reported to predict treatment outcomes in various cancer entities [57] and to detect minimal residual disease [58]. Additionally, serial analysis of the cfRNA transcript profile of these genes in metastatic PDAC patients receiving systemic FOLFIRINOX therapy revealed a general increase in cfRNA transcript abundance in post‐treatment samples compared to baseline samples. These findings align with reports indicating that chemotherapy treatment with FOLFIRINOX leads to a subtype switch toward the basal‐like phenotype in pancreatic cancer [17, 20].

Further characterization of *KDELC1* revealed that the expression of this gene was predominantly localized in the stromal compartment of the tumor. Tumors expressing high levels of *KDELC1* exhibited increased stroma content with activated stromal cells, as evidenced by the overexpression of *FAP* and *ACTA2*. Additionally, *KDELC1* ^*high*^ tumors were associated with robust inflammatory processes. This is consistent with literature as activated stroma has been reported to be linked with basal‐like PDAC tumors and tumor aggressiveness in other cancer entities [11, 59]. Moreover, we observed a robust immune infiltration in *KDELC1* ^*high*^ tumors and activation of several inflammatory pathways. The implication of a pro‐inflammatory microenvironment in basal‐like tumors has already been reported [44]. Furthermore, inflammation has been demonstrated to be associated with cancer aggressiveness, and basal‐like PDAC tumors are known to exhibit a high level of aggressiveness [60]. Proteomic analysis of serum samples from PDAC patients with well‐characterized tumor subtypes revealed that several keratins, including KRT2, KRT5, KRT10, and KRT77, were highly abundant in samples from patients with basal‐like tumors. Multi‐omic biomarker panels have been demonstrated to possess stronger diagnostic value compared to single markers [61]. We investigated the predictive values of individual markers and their combinations in predicting tumor subtype. Combinations of different cfRNA transcripts, as well as the KRT77 serum levels, demonstrated a stronger predictive value for tumor subtype compared to individual markers. Lastly, *KDELC1* ^*high*^ and *DEGS1* ^*high*^ tumors exhibited very poor overall survival and resistance to systemic chemotherapy, confirming the association between both markers and basal‐like tumors. Basal‐like tumors are generally characterized by high aggressiveness, leading to very poor overall survival, both in PDAC and other cancer entities [9, 11, 62]. Elevated serum levels of *KDELC1* were similarly associated with worse progression‐free survival and, in a limited number of samples, treatment failure.

## 5 Conclusion

Our proof‐of‐concept study is limited by the number of paired tumor/liquid samples analyzed and the inherent heterogeneity associated with metastatic PDAC. This situation may influence subtype classification, as reported previously [63]. Hence, further prospective studies are warranted. Nonetheless, our findings suggest that a combination of cfRNA and proteomic markers may enable a clinically applicable determination of the molecular tumor subtype and monitoring of possible disease recurrence in a minimally invasive process. In addition, our suggested cfRNA markers potentially facilitate disease detection, monitoring of minimal residual disease, and prediction of treatment response.

## Conflict of interest

MM reports honoraria for advisory boards from Astra Zeneca, Amgen, Boehringer Ingelheim BMS, MSD, Novartis, Roche and Takeda; all have no relation to the submitted work. JTS receives honoraria as consultant or for continuing medical education presentations from AstraZeneca, Bayer, Boehringer Ingelheim, Bristol‐Myers Squibb, Immunocore, MSD Sharp Dohme, Novartis, Roche/Genentech, and Servier. His institution receives research funding from Abalos Therapeutics, AstraZeneca, Boehringer Ingelheim, Bristol‐Myers Squibb, Celgene, Eisbach Bio, and Roche/Genentech; he holds ownership in FAPI Holding (< 3%); all outside the submitted work. The authors declare no conflict of interest.

## Author contributions

Acquired funding and designed the study (MM and SSL); wrote and edited the manuscript (MM, GZ, SSL & HS); provided samples, read and corrected the manuscript, and other resources infrastructure (NN, AM, JJK, VK, TMR, JTS, JDH, SG, AD, SR, PAH and GO'K, GZ, HS); performed experiments and Analyses (SSL, MT‐A & PC).

### Peer review

The peer review history for this article is available at https://www.webofscience.com/api/gateway/wos/peer‐review/10.1002/1878‐0261.13747.

## Supporting information


**Fig. S1.** The cfRNA subtype marker KDELC1 and PTTG2 are over expressed in tissue samples pancreatitis and pancreatic cancer.
**Fig. S2.** Overexpression of *KDELC1* and *DEGS1* is not associated with response to GnP in the COMPASS cohort.
**Fig. S3.** Compartmentalized proteomic analysis of KDELC1 expression in PDAC tumor samples by means of multiplex immunofluorescence.

## Data Availability

All data relevant to this study are already deposited in public repositories and access will be granted upon reasonable request to the study leaders. The data from the clinical trial are available from the corresponding study leaders. All other data are publicly available under the accession number (E‐MTAB‐1791).
