# Supplementary material for: Minimally invasive determination of pancreatic ductal adenocarcinoma (PDAC) subtype by means of circulating cell‐free RNA
Source: Mol Oncol. 2024 Oct 31;19(2):357–76. doi: 10.1002/1878-0261.13747 (PMC11792997; doi:10.1002/1878-0261.13747)
Supplement: Supplementary file 1 — Fig. S1. The cfRNA subtype marker KDELC1 and PTTG2 are over expressed in tissue samples pancreatitis and pancreatic cancer. Fig. S2. Overexpression of KDELC1 and DEGS1 is not associated with response to GnP in the COMPASS cohort. Fig. S3. Compartmentalized proteomic analysis of KDELC1 expression in PDAC tumor samples by means of multiplex immunofluorescence. [file MOL2-19-357-s001.zip › caption_Supplementary data.docx]

***Supplementary information***

Supplementary figures 1 - 3

**Figure S1: The cfRNA subtype marker KDELC1 and PTTG2 are over expressed in tissue samples pancreatitis and pancreatic cancer.** A violin plot presenting the expression pattern of *KDELC1* in non-tumor pancreas, pancreatitis and pancreatic cancer tissue (left panel). A violin plot presenting the expression pattern of *PTTG2* in non-tumor pancreas, pancreatitis and pancreatic cancer tissue (right panel). Presented p values are obtained from a Mann-Whitney test.

**Figure S2: Overexpression of *KDELC1* and *DEGS1* is not associated with response to GnP in the COMPASS cohort.** Waterfall plots show the distribution of the best overall response to treatments in patients within the COMPASS cohort. Patients are dichotomized to either *KDELC1^high^* and *KDELC1^low^* expression tumor tissue (A) or *DEGS1^high^* and *DEGS1^low^* (B) respectively. Dichotomization is based on a predetermined cut-off defined in the Cox proportional hazards analysis.

**Figure S3: Compartmentalized proteomic analysis of KDELC1 expression in PDAC tumor samples by means of multiplex immunofluorescence.** A) Representative images of KDELC1 expression in tumors with low (left panel) and high (right panel) stromal content. B) Quantitative analysis of KDELC1 protein expression in tumor and stroma cells.
