# Supplementary figures and images for: Minimally invasive determination of pancreatic ductal adenocarcinoma (PDAC) subtype by means of circulating cell‐free RNA
Source: Mol Oncol. 2024 Oct 31;19(2):357–76. doi: 10.1002/1878-0261.13747 (PMC11792997; doi:10.1002/1878-0261.13747)

*KDELC1*

*PTTG2*

$P < 0.0001$

$P = 0.0010$

$P < 0.0001$

$P = 0.0237$

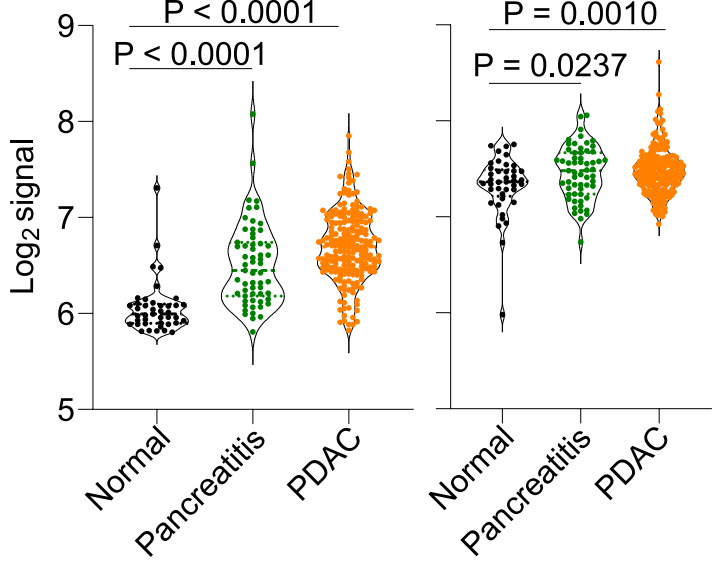

Supplement: Supplementary file 1 — Fig. S1. The cfRNA subtype marker KDELC1 and PTTG2 are over expressed in tissue samples pancreatitis and pancreatic cancer. Fig. S2. Overexpression of KDELC1 and DEGS1 is not associated with response to GnP in the COMPASS cohort. Fig. S3. Compartmentalized proteomic analysis of KDELC1 expression in PDAC tumor samples by means of multiplex immunofluorescence. [file MOL2-19-357-s001.zip › mol213747-sup-0001-FigureS1.pdf]

(A)

COMPASS GnP cohort (N = 83)

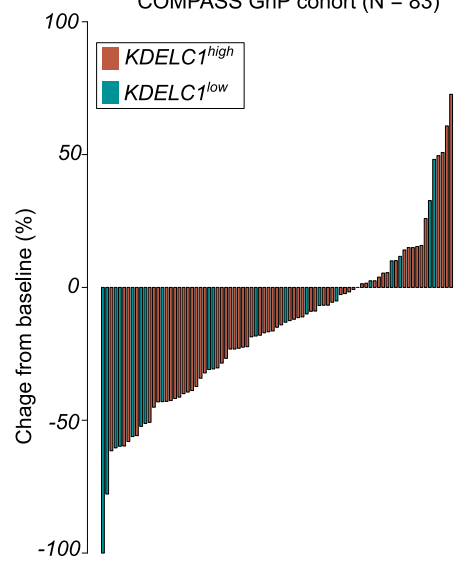

(B)

COMPASS GnP cohort (N = 83)

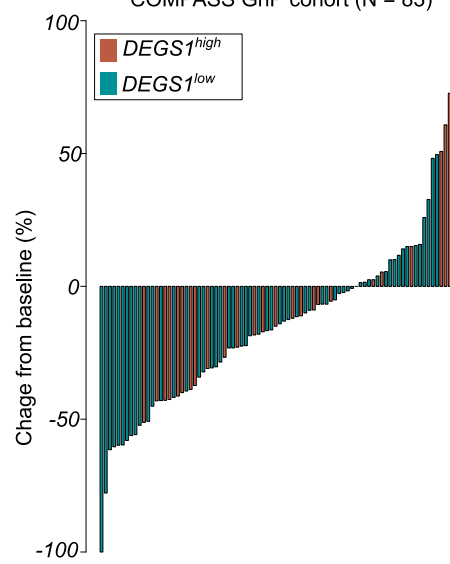

Supplement: Supplementary file 1 — Fig. S1. The cfRNA subtype marker KDELC1 and PTTG2 are over expressed in tissue samples pancreatitis and pancreatic cancer. Fig. S2. Overexpression of KDELC1 and DEGS1 is not associated with response to GnP in the COMPASS cohort. Fig. S3. Compartmentalized proteomic analysis of KDELC1 expression in PDAC tumor samples by means of multiplex immunofluorescence. [file MOL2-19-357-s001.zip › mol213747-sup-0002-FigureS2.pdf]

**(A)**

Low stromal content

High stromal content

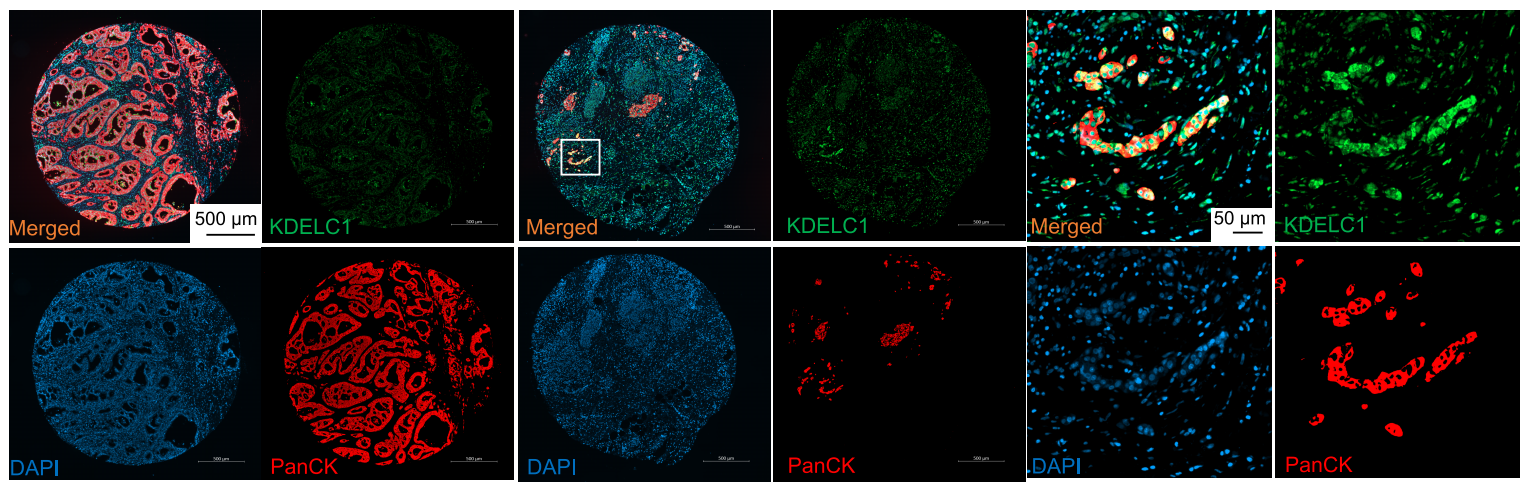**(B)**

%Positive\_stroma

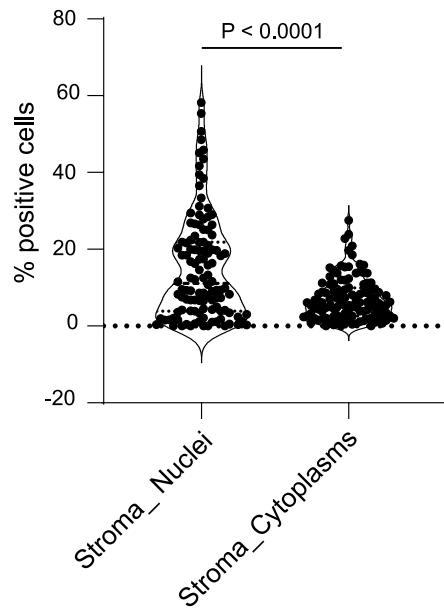

%Positive\_Tumor

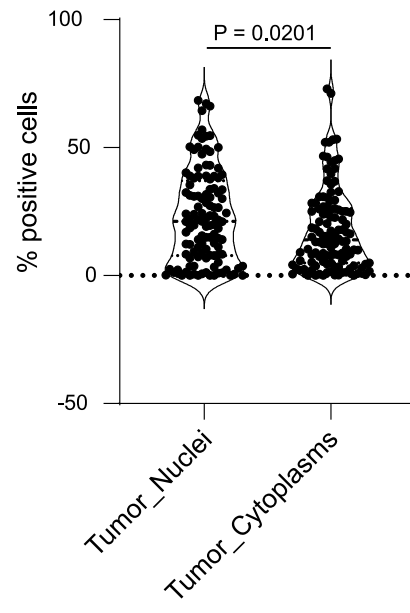

Supplement: Supplementary file 1 — Fig. S1. The cfRNA subtype marker KDELC1 and PTTG2 are over expressed in tissue samples pancreatitis and pancreatic cancer. Fig. S2. Overexpression of KDELC1 and DEGS1 is not associated with response to GnP in the COMPASS cohort. Fig. S3. Compartmentalized proteomic analysis of KDELC1 expression in PDAC tumor samples by means of multiplex immunofluorescence. [file MOL2-19-357-s001.zip › mol213747-sup-0003-FigureS3.pdf]
